# Quantifying VMAT2 target occupancy at effective valbenazine doses and comparing to a novel VMAT2 inhibitor: a translational PET study

**DOI:** 10.1038/s41386-024-02046-3

**Published:** 2025-01-05

**Authors:** Ryan Terry-Lorenzo, Daniel Albrecht, Sabrinia Crouch, Richard Wong, Gordon Loewen, Nagdeep Giri, Heather Skor, Kelly Lin, Christine M. Sandiego, Meghan Pajonas, Eugenii A. Rabiner, Roger N. Gunn, David S. Russell, Dietrich Haubenberger

**Affiliations:** 1 Neurocrine Biosciences, Inc., San Diego, CA USA; 2 Invicro, LCC., Needham, MA USA

**Keywords:** Medical research, Transporters in the nervous system

## Abstract

Positron emission tomography (PET) is frequently used to obtain target occupancy (%TO) of central nervous system (CNS) drug candidates during clinical development. Obtaining %TO with PET can be particularly powerful when the %TO associated with efficacy is known for a protein target. Using the radiotracer [^18^F]AV-133, the relationship between plasma concentration (PK) and %TO of NBI-750142, an experimental inhibitor of the vesicular monoamine transporter type 2 (VMAT2) was obtained in both nonhuman primate (NHP) and human. This work established [^18^F]AV-133 PET as capable of providing a VMAT2 inhibitor PK-%TO relationship that translated from NHP to human. To establish the VMAT2%TO benchmark, PET was performed in NHP with NBI-98782, the main active metabolite of valbenazine, and this PK-%TO relationship was used to estimate VMAT2%TO at NBI-98782 exposures associated with valbenazine therapeutic effects in the treatment of tardive dyskinesia (TD). This work defined 85–90% as the VMAT2%TO achieved by exposures associated with daily dosing with 80 mg valbenazine, a dosing regimen known to exhibit a large effect size in the treatment of TD and in the treatment of chorea associated with Huntington’s Disease. NBI-750142 was estimated to achieve 36–78% VMAT2 target occupancy at acceptable doses, indicating potential inferiority in conferring clinical benefit compared to valbenazine. It is recommended that the %TO benchmark of valbenazine derived from [^18^F]AV-133 PET serve as a gold standard biomarker to evaluate novel VMAT2 inhibitors undergoing clinical development.

## Introduction

Positron Emission Tomography (PET) is an invaluable tool to obtain target occupancy (%TO) for drug candidate compounds [1, 2]. Because PET is precise and relatively non-invasive, it is one of the few technologies that quantifies drug candidate target occupancy in the living brain, informing Go/No-Go and dose selection decisions. Although the desired %TO to deliver therapeutic benefit often is unknown, for drugs with proven clinical efficacy, PET can quantify %TO at doses established to be efficacious in treating human illness. An example would be dopamine receptor (D2R) antagonists, which are effective antipsychotics. First-generation antipsychotics were developed decades ago, largely using rodent pharmacology [3]. After further understanding of antipsychotic molecular target(s) [4], and validated PET radioligands development [5], antipsychotic D2R %TO was determined. This body of work defined antipsychotic D2R %TO leading to therapeutic benefit (65–80%), with higher %TO leading to extrapyramidal and other adverse events (>80%) [6, 7], although refinements to these benchmarks continue as more data emerge [8].

In this study, the %TO of NBI-750142, an experimental VMAT2 (vesicular monoamine transporter type 2) inhibitor, was established in nonhuman primate (NHP) and humans, using the validated VMAT2 radiotracer, [^18^F]AV-133 [9]. Having established NHP-human [^18^F]AV-133 PET translatability, NHP PET was used to estimate the %TO needed for effective treatment of tardive dyskinesia (TD) by valbenazine, an FDA-approved VMAT2 inhibitor. VMAT2 is a brain-specific transporter responsible for packaging monoamine neurotransmitters from the cytosol into the synaptic vesicle lumen, preparing neurotransmitters for release into the synaptic cleft [10]. VMAT2 inhibitors reduce monoamine packaging, leading to reduced monoamine release and neurotransmission [11]. Among these monoamines is dopamine, and dysregulated dopamine signaling contributes to several movement disorders, including TD [12, 13]. TD is a debilitating movement disorder associated with prolonged use of dopamine receptor-blocking agents, such as antipsychotics, and is characterized by uncontrolled movements, particularly in the orofacial area [14]. VMAT2 inhibitors are effective TD treatments [15, 16], and evidence suggests the primary mechanism for efficacy is inhibition of VMAT2-dependent vesicular dopamine transport [13].

Valbenazine is the valine ester of (+)-α-dHTBZ (dihydrotetrabenazine); in humans, valbenazine is converted to [+]-α-dHTBZ [17] (referred to as “NBI-98782” in this paper). While tetrabenazine and deutetrabenazine are converted into four dHTBZ enantiomers with variable VMAT2 inhibitor potencies and off-target activities, NBI-98782 has nanomolar potency at VMAT2, with minimal to non-detectable inhibition of other targets [17–19]. Thus, valbenazine treatment in humans is expected to lead to specific VMAT2 inhibition.

KINECT 3, a pivotal Phase III trial in TD patients [15], demonstrated the efficacy and tolerability of valbenazine 40 mg and 80 mg as evidenced by reduction in AIMS (Abnormal Involuntary Movement Scale) score after 6 weeks of once daily oral administration. As demonstrated in KINECT 3, valbenazine 40 mg effectively treated TD (Cohen’s *d* = 0.5), while valbenazine 80 mg led to a large effect size in treating TD (Cohen’s *d* = 0.9). Using a reduction of >50% from baseline in the AIMS score as a cut-off for identifying patients with a robust dyskinesia treatment response, KINECT 3 showed that 9% of placebo-treated TD patients, 24% of patients on valbenazine 40 mg, and 40% of patients on valbenazine 80 mg had a robust AIMS response after 6 weeks of treatment [15]. A Phase III trial in chorea associated with Huntington’s Disease further confirmed the efficacy of valbenazine in the treatment of an additional movement disorder [20] (valbenazine 80 mg: Cohen’s *d* = 0.9). While previous estimates of %TO at these doses were extrapolated from molecular pharmacology data [21], this study is the first to use PET to define VMAT2%TO at drug exposures matched to these efficacious valbenazine doses. Our goals in this study were (1) to establish a translational NHP—human PET platform to determine exposure-%TO relationships for VMAT2 inhibitors, and (2) use that platform to benchmark VMAT2%TO at therapeutic doses of valbenazine to enable comparison to other VMAT2 inhibitors.

## Materials and methods

### Nonhuman primate PET

The NHP studies were conducted in full compliance with either the Yale University or Charles River Laboratories Institutional Animal Care and Use Committee (IACUC) policies and procedures, which follow the recommendations of The Guide for the Care and Use of Laboratory Animals (“The Guide”, Institute of Laboratory Animal Resources, National Academy Press, Washington, D.C., 8th Edition, 2011). Each sites’ animal programs are accredited by AAALAC International.

In total, three NHP PET studies were conducted using the following VMAT2 inhibitors as blocking compounds: NBI-750142, NBI-98782, and NBI-751508. Each NHP PET study enrolled two to three cynomolgus macaques, (3.8–9 year age range).

NHPs fasted overnight before individual PET scans. For the NBI-750142 study, 60 min prior to NBI-750142 or vehicle administration, animals were sedated with an intramuscular injection of 2 mg/kg Aflaxan, 0.02 mg/kg dexmedetomidine, and 0.3 mg/kg midazolam. The animals were intubated with an endotracheal tube for continued delivery of oxygen (1.5–2.5 L) and 1.0–2.5% isoflurane for anesthesia maintenance.

For the two other studies, prior to administration of NBI-751508, NBI-98782, or vehicle, animals were sedated with 5–10 mg/kg intramuscular ketamine. The animals were intubated with an endotracheal tube for continued delivery of isoflurane for anesthesia maintenance.

For all NHP PET scans, blocking compound was administered with a bolus loading dose, followed by constant intravenous infusion over 3 h, with the goal of achieving steady-state plasma exposure. One hour after blocking compound or vehicle administration, [^18^F]AV-133 was administered as an intravenous bolus. Dynamic PET data were acquired with a MicroPET Focus-220 camera (Siemens Microsystems, Knoxville, TN) for 120 min post injection and reconstructed into a series of 33 frames and all standard corrections were applied for normalization, randoms, scatter, and attenuation. Brain PET images were analyzed using PMOD software package v3.802 (PMOD Technologies, Zurich, Switzerland). PET images were rigidly aligned to the NHP’s own structural T1 MRI and then spatially normalized to a common cynomolgus MRI template for anatomical brain region definition.

During each scan, 1 mL whole blood PK samples were collected (in K2 EDTA tubes) at multiple time points. Blocking compound concentration in plasma samples were analyzed using validated LC-MS/MS bioanalytical methods. Plasma samples were processed using liquid-liquid extraction and the addition of stable isotope-labeled internal standard. Processed samples were chromatographically separated on a reversed-phase C18 column and coupled to a Sciex API 4000 or Sciex API 5000 triple quadrupole mass spectrometer. The average plasma concentration, C_ave,_ was calculated as the mean compound concentration of samples collected during the PET scan and used for PK-TO analysis.

Time-activity curves were generated for the caudate, putamen, and cerebellum. Non-displaceable binding potential (BP_ND_) was estimated using non-invasive Logan graphical analysis with cerebellum as reference region. %TO of the blocking compound was calculated in the caudate and putamen using baseline and post-dose BP_ND_ as follows:

1 $$\% {TO}=1-\frac{{{BP}}_{{ND}}\left({post}-{block}\right)}{{{BP}}_{{ND}}\left({baseline}\right)}x100$$

The relationship between %TO (averaged between caudate and putamen) and C_ave_ was fit with a simplified E_max_ model:

2 $$\% {TO}=\frac{[{Drug}]}{\left[{Drug}\right]+\,{{EC}}_{50}}$$

Free EC_50_ (concentration needed to achieve half-maximal %TO) was derived by accounting for cynomolgus monkey plasma protein binding (PPB).

The two NHPs for the NBI-98782 study were re-enrolled into the NBI-751508 study, allowing for a test-retest analysis to be performed on baseline scans conducted ~5 months apart. Test-retest variability of BP_ND_ in the caudate and putamen were calculated as

3 $${TRTV}\left( \% \right)=100\,x\,\frac{{{BP}}_{{ND}}^{1}-{{BP}}_{{ND}}^{2}}{({{BP}}_{{ND}}^{1}{+{BP}}_{{ND}}^{2})/2}$$

### Human PET

The human PET study was approved by the IRB (Approval number: Pro00037529). All participants gave their written informed consent prior to participation in the study. Twelve healthy male subjects were enrolled in cohorts of 2–4 subjects each. Subjects were 29–54 years of age (mean of 43.2 years), and the study had standard inclusion/exclusion criteria, with attention to avoiding drugs which may interfere with the [^18^F]AV-133 measurements or with NBI-750142 metabolism.

Subjects fasted during the study and received a small, predefined snack at least 2 h prior to oral dosing of NBI-750142. Consumption of alcohol, caffeine, nicotine or cannabis products, and grapefruit or grapefruit juice was prohibited during the study. One PET scan occurred ~1.5 h following administration of NBI-750142, and a second ~18 h post NBI-750142. These scans are referred to as “T1” and “T2,” respectively, throughout this study (Supplementary Fig. 1). T1 scans were timed to occur at approximately the time to reach maximal plasma concentration, *T*_max_, and provide the closest comparison to the NHP PET measurements. T2 scans should contribute data points to the lower NBI-750142 concentration portion of the PK-%TO curve and potentially reveal dose-response hysteresis (reviewed in ref. [22]). A total of five cohorts were enrolled in the study in an adaptive design, with two subjects enrolled in each cohort, except for four subjects in cohort 5. NBI-750142 doses in these cohorts ranged from 10 to 200 mg. Blood samples were obtained from each participant immediately before [^18^F]AV-133 injection and just before the end of the PET scan in order to quantify plasma concentration of NBI-750142. The C_ave_ derived from these two values was used to compare against %TO.

Prior to the radiotracer injection and PET, a low-dose CT scan was collected for attenuation correction. Subjects were administered a single dose of [^18^F]AV-133 intravenously over 3 min using an infusion pump (~3.33 mL per min) through a venous catheter followed by a 10 mL saline flush. Dynamic PET imaging of the brain was acquired over 120 min following radiotracer injection on a Siemens Biograph 6 PET/CT camera with the following frame times: 6 × 30 s, 4 × 1 min, 4 × 2 min, 21 × 5 min (0–120 min). Images were reconstructed with a 168 × 168 × 81 matrix (pixel size of 2.03 mm × 2.03 mm) using an iterative reconstruction algorithm (OSEM 4 iterations, 16 subsets) and a post hoc Gaussian filter FWHM = 3 mm. Standard corrections for random, scatter, system dead time, and attenuation provided by the camera manufacturer were performed. A T1 MRI scan was acquired from eligible subjects as part of the screening visit, to identify and delineate brain anatomical regions of interest for individual PET images. MRI scans were obtained on a Siemens Espree 1.5 Tesla clinical magnet.

Reconstructed PET images were motion and decay corrected, realigned to the subject MRI and subsequently normalized into standard MNI (Montreal Neurological Institute) space using PMOD. Volumes of interest (VOIs) were defined from a template (Hammers, N30R83). Average activity concentration within each VOI was determined and time activity curves (TACs) were generated. TACs were extracted from the following VOIs: caudate and putamen (striatum), and occipital cortex. BP_ND_, the primary outcome measure, was estimated with noninvasive Logan graphical analysis [23] (NI-LGA, t* = 20 min) in caudate and putamen using the occipital cortex as the reference region. %TO of NBI-750142 (drug) was computed Eq. 1. The relationship between %TO (averaged between caudate and putamen) and average plasma concentration during the scan was analyzed with an *E*_max_ model (Eq. 2) and an upregulation model (Eq. 4), which incorporates an upregulation factor (URF; [24]).

4 $$\% {TO}=\frac{\left[{Drug}\right]-\,{{EC}}_{50}\frac{\left[{Drug}\right]}{\left[{Drug}\right]+\,{{EC}}_{50}}({URF}-1)}{\left[{Drug}\right]+{{EC}}_{50}}$$

### Matching NHP PET to human PK

Valbenazine human plasma exposure metrics in patients were estimated with a population pharmacokinetic (PK) analysis and plasma protein binding (PPB) data. In this manner, unbound concentration metrics for NBI-98782 at steady-state following 40 mg and 80 mg daily valbenazine doses were determined. These steady-state concentrations, in conjunction with free EC_50_ from NHP PET for blocking compounds was used to estimate %TO in humans. Plasma concentrations of NBI-750142 following 60 mg BID dose were estimated using plasma concentrations following a single oral dose of 60 mg NBI-750142 using the principle of superposition.

### In vitro

The PPB of NBI-98782, NBI-750142, and NBI-751508 was determined in vitro using equilibrium dialysis in pooled plasma from cynomolgus monkeys and humans (mixed gender). *K*_i_ values for compound affinity to VMAT2 were generated from human platelet homogenates as described in [18].

## Results

### Non-human primate NBI-750142 PET study

**Fig. 1:**
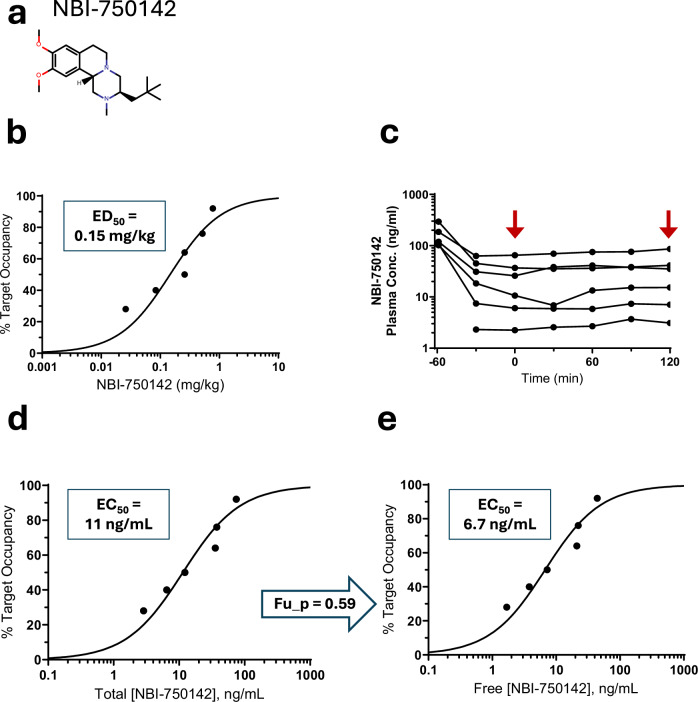
NBI-750142 non-human primate PET. **a** Chemical structure of NBI-750142. **b** Relationship between mg/kg dose of NBI-750142 administered to NHP and matching % target occupancy (%TO). **c** NBI-750142 concentration in plasma measured at multiple time points relative to injection of the [^18^F]AV-133 radiotracer in each individual NHP. Arrows depict the start and finish of the 120-min PET scan. **d** Relationship between average total NBI-750142 concentration and %TO. **e** Relationship between average free NBI-750142 concentration and %TO. Free NBI-750142 was determined by multiplying the total compound concentration by the fraction unbound in plasma (fu_p). In panels **b**, **d**, and **e**, an *E*_max_ model was applied to the data, with the appropriate potency parameter defined in the panel.

**Fig. 2:**
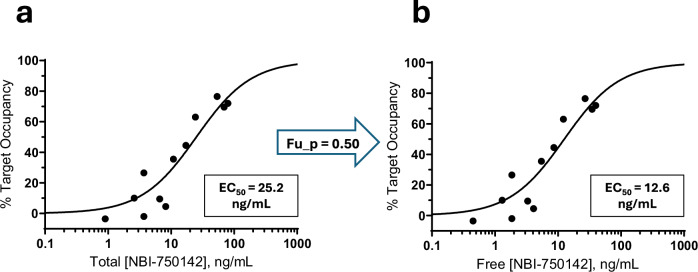
NBI-750142 human PET. **a** Relationship between average total NBI-750142 concentration during the [^18^F]AV-133 PET scan and %TO. **b** Relationship between average free NBI-750142 concentration and %TO. In this figure, only T1 values are depicted. Free NBI-750142 was determined by multiplying the total compound concentration by the fraction unbound in plasma (fu_p). In both panels, an *E*_max_ model was applied to the data, with the EC_50_ potency parameter defined in the panel.

In this study, NBI-750142 bound VMAT2 with a sigmoidal dose-response curve. These data were well-fit by a standard *E*_max_ model to provide a 0.15 mg/kg ED_50_ value (Fig. 1b). NBI-750142 concentration was measured before and throughout the dynamic scan; steady-state was achieved with relatively stable NBI-750142 plasma concentrations during the PET scan (Fig. 1c). The mean NBI-750142 concentration during the 120-min PET scan (C_ave_) was matched to target occupancy in each PET scan to produce a relationship between compound concentration and %TO (Fig. 1d). These data were well-fit by a standard *E*_max_ model to provide an 11 ng/mL EC_50_ value (Fig. 1d). Because of near linear pharmacokinetics, Fig. 2b, d appear nearly identical, despite different *x*-axis values. The measured fraction unbound in plasma (fu_p) value of 0.59 was used to convert total NBI-750142 concentration on the *x*-axis of Fig. 1d to free NBI-750142 concentration (Fig. 1e) to produce the free EC_50_ of 6.7 ng/mL.

### Human NBI-750142 PET study

We subsequently assessed the translatability of NHP PET outcomes to human participants by performing a comprehensive dose-response NBI-750142 PET study in healthy volunteers, using NBI-750142 doses considered safe and tolerable in prior studies. The T1 occupancy values from the human PET study were well-fit by an *E*_max_ model and produced a total NBI-750142 EC_50_ of 25.2 ng/mL (Fig. 2a) and a free NBI-750142 EC_50_ of 12.6 ng/mL (Fig. 2b). This free EC_50_ is within twofold of the 6.7 ng/mL NHP EC_50_ (Fig. 1e); even this minimal difference is partially explained by slightly lower potency of NBI-750142 in binding human versus NHP VMAT2 in in vitro experiments, such that the EC_50_:*K*_i_ ratios for NBI-750142 in both species is very nearly 1 (Table 1). The addition of T2 occupancy values, however, distorted the curve (Supplementary Fig. 2A), notably influenced by several “negative occupancy” values in which post-dose BP_ND_ was increased compared to baseline. Negative occupancy observed at T2 (i.e., higher BP_ND_ at T2 compared with baseline) may be explained by “upregulation” of VMAT2 post NBI-750142 after some time (see Discussion). Thus, a recently developed upregulation model [24], which adds an URF parameter, was applied to both T1 and T2 data, generating an EC_50_ of 6.3 ng/mL and a URF of 2.8 (Supplementary Fig. 2B). The Akaike Information Criteria (AIC) value can be used to compare the quality of models to fit experimental data, even when the models have different numbers of adjustable parameters [25]. When fit to the entire data set (T1 combined with T2 values), AIC for the upregulation model was lower than the *E*_max_ model (Supplementary Table 1), demonstrating that the upregulation model better fit the entire dataset. The upregulation finding was further explored with additional post hoc analyses (see Supplementary section).

**Table 1:** Summary of free EC_50_ values derived from PET, molecularly derived Ki values, and ratios of the two values for each VMAT2 inhibitor compound.

Compound	Species	EC_50_ (ng/mL)	*K*_i_ (ng/mL)	EC_50_:*K*_i_ Ratio
NBI-750142	NHP	6.7	6.1	1.1
NBI-750142	Human	12.8	9.2	1.4
NBI-98782	NHP	1.5	3.9	0.4
NBI-751508	NHP	1.1	1.8	0.6

*EC*_*50*_ free compound concentration at 50% target occupancy (from PET experiments), *Ki* inhibitor constant measured in vitro, utilizing purified protein from species of interest, *NHP* nonhuman primate, *VMAT2* vesicular monoamine transporter 2.

### Non-human primate NBI-98782 PET study

**Fig. 3:**
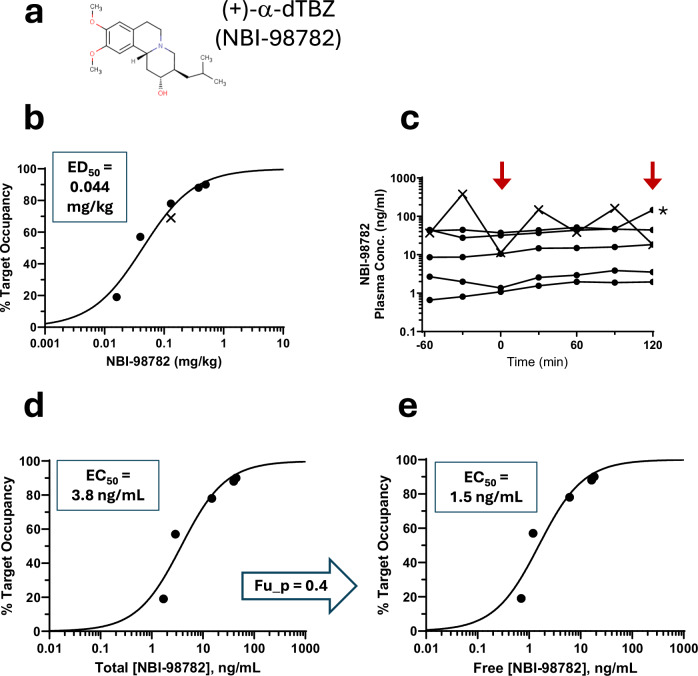
NBI-98782 non-human primate PET. **a** Chemical structure of NBI-98782. **b** Relationship between mg/kg dose of NBI-98782 administered to NHP and matching % target occupancy (%TO). **c** NBI-98782 concentration in plasma measured at multiple time points relative to injection of the [^18^F]AV-133 radiotracer in each individual NHP. Arrows depict the start and finish of the 120-min PET scan. The dose depicted by the “x” symbol was highly variable over time. Because there was no explanation for this phenomenon, this data point was not used in panels d and e. Likewise, due to unexplained deviation from other measures in the same scan, the data point depicted with an asterisk was not included in determining the mean ng/mL NBI-98782 concentration (C_ave_) during that scan. **d** Relationship between average total NBI-98782 concentration and %TO. **e** Relationship between average free NBI-98782 concentration and %TO. Free NBI-98782 was determined by multiplying the total compound concentration by the fraction unbound in plasma (fu_p). In panels **b**, **d**, and **e**, an *E*_max_ model was applied to the data, with the appropriate potency parameter defined in the panel.

While the NBI-750142 PET studies enable conversion of doses or exposures of NBI-750142 into estimations of %TO, alone these data do not define the desired %TO to achieve therapeutic goals. To provide that guidance, an NHP PET study was conducted using NBI-98782 (Fig. 3a), the active metabolite of valbenazine. These data were well-fit by a standard *E*_max_ model to provide a 0.044 mg/kg ED_50_ value (Fig. 3b). NBI-98782 PK data (Fig. 3c) were matched to each PET scan to produce a relationship between compound plasma concentration and %TO (Fig. 3d). A standard *E*_max_ model provided an EC_50_ value of 3.8 ng/mL. The in vitro fraction unbound in plasma (fu_p) value of 0.4 was used to convert total NBI-98782 concentration on the *x*-axis of Fig. 1c to free NBI-98782 concentration (Fig. 3e) to produce the free EC_50_ of 1.5 ng/mL.

### Benchmarking valbenazine VMAT2 target occupancy at efficacious doses and comparing to NBI-750142 target occupancy

With several assumptions (see Discussion), the 1.5 ng/mL NBI-98782 NHP PET EC_50_ value could be used to convert free (unbound) NBI-98782 concentration (PK), measured in plasma, to an estimated %TO. For this analysis, three plasma PK parameters, *C*_max_ (maximal concentration), *C*_ave_ (average concentration) and *C*_trough_ (lowest concentration), were modeled at steady state in TD patients receiving daily, efficacious doses of valbenazine. As shown in Table 2, this analysis demonstrated that patients taking the 40 mg dose of valbenazine have VMAT2%TO estimations between 73% and 82%. Patients taking the 80 mg dose of valbenazine have VMAT2%TO estimations between 85% and 91%.

**Table 2:** Using NHP PET to convert human plasma PK parameters into VMAT2% target occupancy estimations.

*ng/mL Total*^a^				*ng/mL Free*^b^				*VMAT2%TO*^c^			
Dose	*C*_max_	*C*_avg_	*C*_trough_	Dose	*C*_max_	*C*_avg_	*C*_trough_	Dose	*C*_max_	*C*_avg_	*C*_trough_
40 mg VBZ, QD	19.2	16.1	11.0	40 mg VBZ, QD	6.9	5.8	4.0	40 mg VBZ, QD	82%	79%	73%
80 mg VBZ, QD	40.0	32.3	22.9	80 mg VBZ, QD	14.4	11.6	8.2	80 mg VBZ, QD	91%	89%	85%
60 mg NBI-750142, BID	48.1	27.7	7.6	60 mg NBI-750142, BID	24.1	13.9	3.8	60 mg NBI-750142, BID	78%	67%	36%

*%TO* target occupancy, *BID* twice-daily dosing, *C*_avg_ average concentration, *C*_max_ maximal concentration, *C*_trough_ lowest concentration, *QD* once-daily dosing, *VMAT2* vesicular monoamine transporter 2.

^a^Pharmacokinetic (PK) parameters for NBI-98782 (active metabolite of VBZ) at steady-state listed in the table were determined by a population PK analysis using patient data.

^b^Free compound concentration is determined by multiplying total concentration by fraction unbound in plasma (fu_p).

^c^Target occupancy (%TO) was determined by converting free compound concentration into %TO, using the appropriate NHP PET EC_50_ (Figs. 1e and 3e) for each compound.

Using the above strategy, any tetrabenazine-analog VMAT2 inhibitor drug candidate could be compared to these %TO benchmarks. To validate the hypothesis, %TO values were derived for NBI-750142. To minimize differential variables, the 6.7 ng/mL free EC_50_ from the NHP NBI-750142 study was used for this comparison. The human PK plasma concentration following 60 mg twice daily (BID) dosing of NBI-750142, the highest dose estimated to be reasonably and safely administered in multi-day studies in human patients, was estimated from human data following a single oral dosing of 60 mg NBI-750142. As shown in Table 2, this analysis demonstrated that the expected plasma concentration of NBI-750142, following administration of 60 mg BID, results in VMAT2%TO estimations between 36% and 78% which were lower than the 40 mg valbenazine dose.

To provide additional data to assess our VMAT2 PET platform, an NHP PET study was performed with the novel VMAT2 inhibitor, NBI-751508. The NBI-751508 chemical structure and NHP PET data are presented in Supplementary Fig. 3. NBI-751508 NHP PET free EC_50_ was 1.1 ng/mL (Table 1). During the implementation of this study, baseline [^18^F]AV-133 PET scans were reassessed in NHPs rolled over from the NBI-98782 study, demonstrating high test-retest precision in these 2 NHPs (<10% in caudate and putamen; Supplementary Table 2).

## Discussion

### VMAT2 occupancy required for efficacy

Our study suggests that high, sustained VMAT2 occupancy is required for optimal TD treatment. After reaching steady state, 80 mg valbenazine is estimated to have ~90%TO, remaining above 85%TO even at C_trough_; 40 mg valbenazine is estimated to have ~80% average %TO. As pivotal, well-powered, placebo-controlled studies have demonstrated efficacy with both 40 and 80 mg valbenazine [15], the collective evidence is that 80–90% sustained occupancy of VMAT2 is required for TD treatment that is both safe and optimally effective. Thus, while it appears that the treatment of TD by valbenazine follows a monotonic dose-response curve in which the highest tolerable dose provides the greatest efficacy, both 40 and 80 mg doses are appropriate in effectively treating TD, and dosing can be adjusted based on patient response and tolerability. Valbenazine is also available in 60 mg doses for further customization.

This level of VMAT2 occupancy at tolerable, efficacious doses is higher than what has been observed for D2R antagonist antipsychotics. Antipsychotic therapeutic efficacy occurs at ~65% occupancy while abnormal movements and other adverse events occur at >80% occupancy [6–8]. Target occupancies more quantitatively comparable to what we observed in our analysis with VMAT2 are found in studies of SERT (plasma membrane serotonin transporter), which is in the same solute carrier (SLC) class as VMAT2 [26]. Selective serotonin reuptake inhibitors (SSRIs), which treat major depressive disorder by inhibiting SERT, display SERT occupancy of 80% at minimally efficacious doses [27] with 85% SERT occupancy at higher doses often used clinically [28]. Because several SSRIs have relatively long-half-lives [29], once-daily SSRI dosing would be expected to engage the SERT target at a high and sustained level throughout the day, as seen for the optimal and sustained valbenazine occupancy of VMAT2 reported here.

### Assumptions of this study and comparing to published work

Comparing to published findings, the NBI-98782 dose response in Fig. 3b is similar to an NHP experiment reported by Kilbourn et al. [30]. Although the radioligand used is different ([^18^F]AV-133 vs. [+]-α-[^11^C]dHTBZ), and the NBI-98782 administration protocol is different (bolus-infusion vs. bolus), our 0.044 mg/kg ED_50_ is nearly identical to the 0.055 mg/kg ED_50_ reported by Kilbourn et al. [30]. In our study, we further collected plasma compound concentrations during the dynamic PET scans to obtain PK-%TO relationships to facilitate comparison across species. The conclusions drawn from this approach required several assumptions: (1) A stable [^18^F]AV-133 baseline facilitates a baseline-postdose protocol; (2) all inhibition of VMAT2 by valbenazine is contributed by NBI-98782; (3) the PK-%TO relationship is similar between NHP and human; and (4) free drug hypothesis principles are relevant for valbenazine, NBI-750142, and NBI-98782.

Exploring these four assumptions in order,

1. The test-retest precision of [^18^F]AV-133 [31, 32], and other VMAT2 radioligands [30, 33], is excellent, in the 5–10% range, and our *n* = 2 NHP [^18^F]AV-133 test-retest (Supplementary Fig. 2) is consistent with these values. Further, VMAT2 levels, as measured in rats, are resistant to change induced by VMAT2 inhibitors [34, 35]. Thus, these findings should facilitate obtaining target occupancy using this protocol, similar to baseline-postdose protocols used throughout the PET field. That said, our upregulation finding could complicate interpretation, and that consideration is explored in the following section.
2. Although the valbenazine parent compound inhibits VMAT2, it is ~40-fold less potent than its active metabolite NBI-98782 [18]. Further, when evaluating PK at steady-state and normalizing by PPB, free NBI-98782 concentrations are higher than free concentrations of the valbenazine parent compound by several fold [36, 37]. Finally, rat studies were conducted in which the NBI-98782 PK-%TO curve was measured when rats were administered valbenazine or directly administered NBI-98782. In those two conditions, the NBI-98782 PK-%TO relationship was nearly identical (Data on file), suggesting that the occupancy of VMAT2 by valbenazine treatment was fully accounted for by NBI-98782.
3. Under most conditions, central nervous system PET findings generally translate from monkey to human [38, 39], and our monkey-human comparison of NBI-750142 provides experimental support for the assertion that monkey-human PET translation is appropriate for tetrabenazine analog VMAT2 inhibitors.
4. Prior to any comparison between monkey and human, we used PPB data to convert total compound concentration values to free (unbound) compound concentrations. This strategy implicitly endorses free drug hypothesis principles, in which unbound compound concentration at the site of action is most relevant for driving pharmacology. Because the site of action (brain) is an inaccessible tissue in primates, compound in plasma is used as a proxy. This strategy of using plasma PK to quantitatively drive pharmacodynamic expectations typically is accurate when compounds are soluble, membrane permeable, and are not efflux substrates [40]. Our observations within this study suggest these principles are relevant. As shown in Table 1, with 3 different comparisons in NHP and 1 comparison in human, the potency of the free compound-%TO relationship is very similar, whether potency is measured in vitro with molecular pharmacology techniques (K_i_) or measured in vivo with PET (EC_50_). As corroborating evidence, Stahl [21] used K_i_ values matched to PK data to estimate %TO by 40 mg and 80 mg valbenazine, and those estimations are remarkably similar to the %TO values we report in Table 2. While we minimize assumptions by using in vivo PET EC_50_s to match to PK data for %TO estimation, the Stahl analysis produced similar results [21]. This similarity is likely because, for the tetrabenazine analog compounds, unbound compound concentration in the plasma is an excellent proxy for compound concentration at the site of action (i.e., consistent with the free drug hypothesis [40] and indicative of a compound which freely crosses the blood-brain barrier).

### Potential explanations for negative occupancy and “upregulation”

In our human NBI-750142 human PET study, we observed VMAT2 negative occupancy for many T2 values. While we did not observe the negative occupancy phenomenon in our three different NHP PET studies; we did not conduct delayed PET scans similar to the T2 scans in the NHP PET studies. In the human PET study, negative occupancy seen at the T2 scans occurred at relatively low NBI-750142 concentration after subjects experienced higher NBI-750142 exposure at T1 (Supplementary Fig. 1). Thus, potential explanations could involve acute NBI-750142 pharmacodynamic effects followed by compound washout. The negative occupancy values observed in the current study could have several explanations. One possibility is that an acute increase in VMAT2 protein target concentration would result in a higher measured post-drug BP_ND_. However, an approximately threefold VMAT2 protein concentration increase hours after a single inhibitor dose seems biologically implausible, especially since the concentration of the VMAT2 protein has been shown to not change following acute inhibition in rat [34, 35]. Alternatively, competition with endogenous dopamine may partially contribute to the finding. Dopamine competes with tetrabenazine-analog PET radiotracers for binding to VMAT2 [41, 42]. Thus, NBI-750142-induced depletion of vesicular dopamine at Tmax (during the T1 scan) could persist and remain at T2; this reduction in vesicular dopamine would reduce competition with [^18^F]AV-133, resulting in an increase in the BP_ND_. Another hypothesis involves an increase in the affinity of VMAT2 for [18 F]AV-133 which would lead to an “upregulation” effect indistinguishable from an increase in the expression of the VMAT2 protein. Such an increase in affinity 2–3 fold is more biologically plausible, and could occur due to NBI-750142 inducing VMAT2 to traffic to a different subcellular location where it has a different affinity for [^18^F]AV-133. This phenomenon is known to occur with D2Rs, where D2R agonists alter D2R subcellular location, thereby altering affinity for PET radioligands [43]. Another potential mechanism for a change in affinity of VMAT2 could be via allosteric interaction with an altered dopamine concentration, such as occurs when cAMP elevation enhances affinity of phosphodiesterase proteins for their radioligands in PET studies [44, 45]. At this time, we have no experimental evidence to differentiate among these possibilities. However, exploring these possibilities experimentally could expand our understanding of the biology, and perhaps even the therapeutic opportunities, of VMAT2 inhibition.

The high-quality model fits with *E*_max_ models when excluding T2 values, coupled with the adherence of Ki and EC_50_ values described above, suggest that a focus on the (1) monkey PET and (2) T1 values in human PET enable drug development decisions for VMAT2 inhibitors, even in the face of potential drug-induced target “upregulation.”

### Benchmarking %TO related with clinical efficacy

Developing novel VMAT2 inhibitors could enable deeper exploration of VMAT2 biology and perhaps open novel therapeutic opportunities. In exploring VMAT2 inhibitor development, [^18^F]AV-133 and other VMAT2 PET radioligands are invaluable tools to obtain PK-%TO relationships for drug candidate VMAT2 inhibitors. With this study, exposures associated with efficacy in TD were correlated with VMAT2%TO. It is recommended that these %TO targets, particularly for the highly efficacious 80 mg once daily valbenazine dose, serve as benchmarks for comparison to novel VMAT2 inhibitors. Within this study, it was observed that maximal doses of the experimental VMAT2 inhibitor NBI-750142, estimated to be reasonably and safely administered in human patients, would not achieve the same %TO as valbenazine, and this discovery contributed to discontinuation of NBI-750142 development. One caveat with the benchmarking strategy is that different therapeutic areas may require different doses and %TO. For example, when D2R antipsychotics are used to treat mood disorders, they are used at lower doses than when used for treating psychosis [46]. Thus, the VMAT2 target occupancy benchmark targets of 80–90% described in this report should not be rigid, but instead serve as a base case for consideration in drug development efforts. In the absence of biological insight or clinical data suggesting lower occupancies would be relevant for therapeutic areas other than TD [15] and chorea associated with Huntington’s disease [20], we recommend the 80–90% occupancy achieved by once daily valbenazine treatment serve as the gold standard for comparing to novel VMAT2 inhibitors in development.

## Conclusion

Using the experimental VMAT2 inhibitor, NBI-750142, NHP to human translation of [^18^F]AV-133 PET was established to quantify PK-VMAT2%TO. NHP PET was coupled with human PK to benchmark VMAT2%TO at doses of valbenazine that are efficacious in TD treatment. These benchmarks can be used to compare to novel VMAT2 inhibitors, such as NBI-750142. Future research opportunities could include exploration of the upregulation finding emerging from the human NBI-750142 PET study (see Supplemental Text). This research could include mechanistic studies, but it would also be insightful to determine if upregulation is reproducible, if it occurs with other VMAT2 inhibitors, and if upregulation occurs in nonhuman species under any conditions. Finally, obtaining additional human PET data for valbenazine and/or other clinically utilized VMAT2 inhibitors would continue to confirm conclusions and provide additional insight into NHP to human translation for this class of compounds.

## Supplementary information

Supplementary Information

## Data Availability

The datasets generated and/or analyzed during the current study are available from the corresponding author on reasonable request.
