# Correction: Quantifying VMAT2 target occupancy at effective valbenazine doses and comparing to a novel VMAT2 inhibitor: a translational PET study

**DOI:** 10.1038/s41386-025-02055-w

**Published:** 2025-01-22

**Authors:** Ryan Terry-Lorenzo, Daniel Albrecht, Sabrinia Crouch, Richard Wong, Gordon Loewen, Nagdeep Giri, Heather Skor, Kelly Lin, Christine M. Sandiego, Meghan Pajonas, Eugenii A. Rabiner, Roger N. Gunn, David S. Russell, Dietrich Haubenberger

**Affiliations:** 1 Neurocrine Biosciences, Inc., San Diego, CA USA; 2 Invicro, LCC., Needham, MA USA

**Keywords:** Medical research, Transporters in the nervous system

Correction to: *Neuropsychopharmacology* 10.1038/s41386-024-02046-3, published online 05 January 2025

The article “Quantifying VMAT2 target occupancy at effective valbenazine doses and comparing to a novel VMAT2 inhibitor: a translational PET study”, written by Ryan Terry-Lorenzo, Daniel Albrecht, Sabrinia Crouch, Richard Wong, Gordon Loewen, Nagdeep Giri, Heather Skor, Kelly Lin, Christine M. Sandiego, Meghan Pajonas, Eugenii A. Rabiner, Roger N. Gunn, David S. Russell and Dietrich Haubenberger, was originally published under exclusive license to The Author(s), under exclusive licence to American College of Neuropsychopharmacology. As a result of the subsequent decision to publish the article under the open access model, the article’s copyright notice was changed on 13 January 2025 to © The Author(s) 2025 and the article is now distributed under a Creative Commons Attribution 4.0 International License, which permits use, sharing, adaptation, distribution and reproduction in any medium or format, as long as you give appropriate credit to the original author(s) and the source, provide a link to the Creative Commons licence, and indicate if changes were made.

The images or other third party material in this article are included in the article’s Creative Commons licence, unless indicated otherwise in a credit line to the material. If material is not included in the article’s Creative Commons licence and your intended use is not permitted by statutory regulation or exceeds the permitted use, you will need to obtain permission directly from the copyright holder.

To view a copy of this licence, visit http://creativecommons.org/licenses/by/4.0/. The original article has been corrected.

