# Supplementary material for: Quantifying VMAT2 target occupancy at effective valbenazine doses and comparing to a novel VMAT2 inhibitor: a translational PET study
Source: Neuropsychopharmacology. 2025 Jan 5;50(7):1093–101. doi: 10.1038/s41386-024-02046-3 (PMC12089418; doi:10.1038/s41386-024-02046-3)
Supplement: Supplementary file 1 — Supplementary Information [file 41386_2024_2046_MOESM1_ESM.pdf]

Supplementary Information

Quantifying VMAT2 Target Occupancy at Effective Valbenazine Doses and Comparing to a Novel VMAT2 Inhibitor: A Translational PET Study

Ryan Terry-Lorenzo, PhD[1], Daniel Albrecht, PhD[1], Sabrinia Crouch, MS[1], Richard Wong, PhD[1], Gordon Loewen, PhD[1], Nagdeep Giri, PhD[1], Heather Skor[1], Kelly Lin, PhD[1], Christine Sandiego, PhD[2], Meghan Pajonas[2], Eugenii A. Rabiner, FCPsych SA[2], Roger N. Gunn, PhD[2], David S. Russell, MD, PhD[2], and Dietrich Haubenberger, MD[1]

[1]Neurocrine Biosciences, Inc. San Diego, CA

[2]Invicro, LCC. Needham, MA

Corresponding Author: Ryan Terry-Lorenzo, PhD

12780 El Camino Real, Suite 100

San Diego, CA 92130

(858) 617-7600

rlorenzo@neurocrine.com

Supplementary Results

Using an alternative reference region to further explore the upregulation model

Because of the unusual nature of the upregulation finding in the human NBI-750142 study (Supplemental Figure 2), an additional post hoc image analysis was performed to address potential artifacts. Although in vitro VMAT2 expression in the occipital cortex is significantly lower than that in the striatum [1], even a small amount of displaceable signal in a reference region could affect measured occupancy. White matter (WM) exhibits negligible VMAT2 expression and has previously been suggested as a reference tissue for VMAT2 quantification by PET [2]. Therefore, we explored cerebral WM tissue as a reference region to derive BP_{ND} values and determine a PK-%TO relationship. Dynamic [^{18}F]AV-133 data were fit well with a WM-derived input function, and yielded BP_{ND} and ΔBP_{ND} values that were highly correlated with the occipital lobe-derived values, but numerically higher (data not shown). WM-derived BP_{ND} values from T1 scans were well-fit by an E_{max} model, similarly to occipital cortex-derived BP_{ND} values, giving a total NBI-750142 EC_{50} of 24.8 ng/mL (data not shown). The addition of T2 data points to the model did affect the shape of the curve, although visually much less than for the occipital cortex-derived BP_{ND} analysis (Supplementary Fig 2C and 2D). With WM as reference region, the AIC was very similar for the E_{max} and upregulation models (Supplementary Table 1).

Supplementary Discussion

Reference region effects on negative occupancy and "upregulation"

In our human NBI-750142 human PET study, the total dataset (T1 and T2) appeared to be better fit with an upregulation model than with a parsimonious E_{max} model when using occipital cortex as a reference region. However, when using a WM reference, there was less difference between E_{max} and upregulation models. Although the average striatal occupancy for the T2 time points was close to zero for the WM-derived values (0.09%), several subjects still exhibited negative occupancy values at doses where some positive occupancy would be expected. While this result could reflect normal variability, it is also possible that the negative occupancy reflects a true physiological process that is unaccounted for in our current models. It is also important to note that although WM-derived BP_{ND} values were highly correlated with occipital-derived values, WM has not been previously validated as a reference region to quantify [^{18}F]AV-133 binding using the gold standard arterial input function. Future work with arterial sampling would be useful to characterize the bias associated with a WM reference region approach.

Supplementary Figures

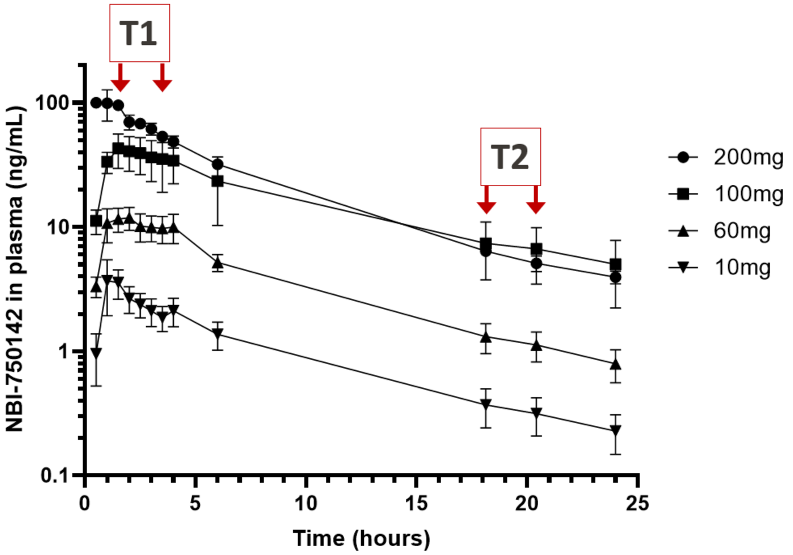

Supplementary Fig. 1 Timing of T1 and T2 PET scans relative to NBI-750142 human pharmacokinetic data. Plasma samples were collected from subjects throughout the PET study after oral administration of NBI-750142, and concentration of NBI-750142 was measured. Data from subjects receiving the same mg dose were pooled and graphed (mean with SEM), with nominal time plotted on the x-axis. Arrows indicate the start and finish of each 2-hour dynamic PET scan. There were two PET scans for each subject at the first (T1) and second (T2) timepoints.

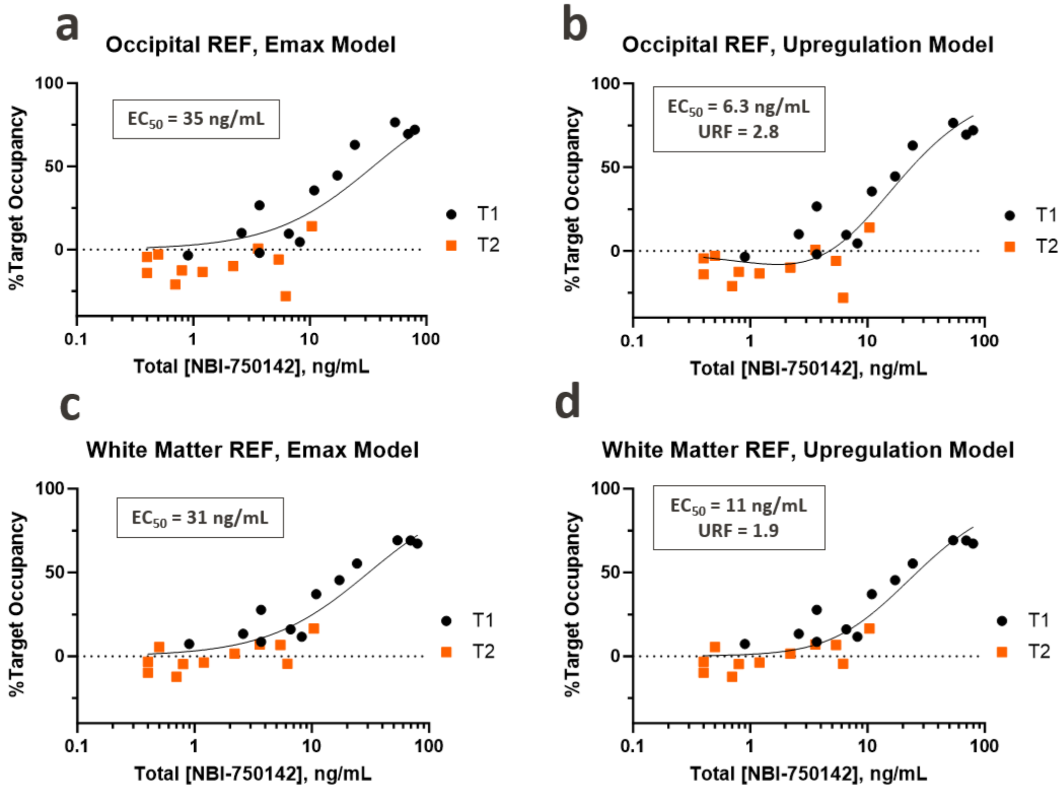

Supplementary Fig. 2 NBI-750142 human PET including both T1 and T2 values. Reanalysis of the NBI-750142 human PET study using two different reference regions (REF) and two different models to fit the PK-occupancy relationship. **a** The occipital lobe reference region, with the Emax model. **b** The occipital lobe reference region, with the upregulation model. **c** The white matter (WM) reference region, with the Emax model. **b** The WM reference region, with the upregulation model. Parameters from the model fits are shown in each panel.

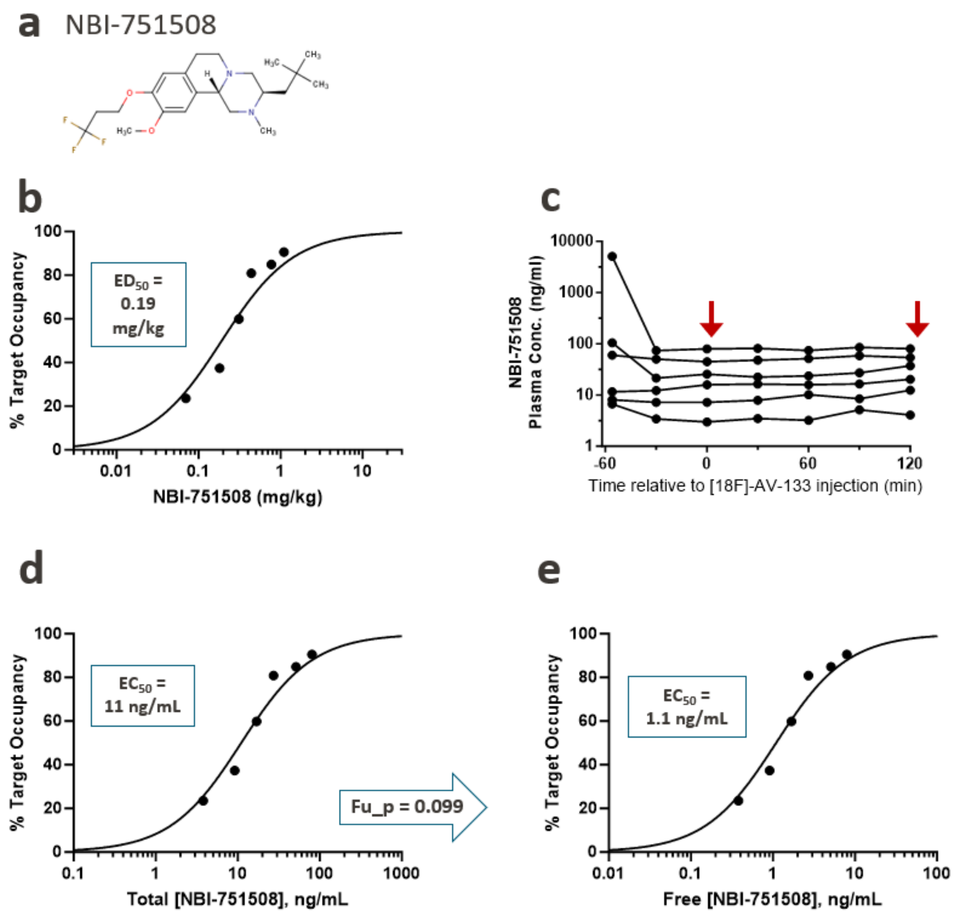

Supplementary Fig. 3 NBI-751508 non-human primate PET. a Chemical structure of NBI-751508. **b** Relationship between mg/kg dose of NBI-751508 administered to NHP and matching % target occupancy (%TO). **c** NBI-751508 concentration in plasma measured at multiple time points relative to injection of the [^{18}F]AV-133 radiotracer in each individual NHP. Arrows depict the start and finish of the 120-minute PET scan. **d** Relationship between average total NBI-751508 concentration and %TO. **e** Relationship between average free NBI-751508 concentration and %TO. Free NBI-751508 was determined by multiplying the total compound concentration by the fraction unbound in plasma (fu_p). In panels b, d, and e, an E_{max} model was applied to the data, with the appropriate potency parameter defined in the panel.

Supplementary Tables

Supplementary Table 1. Model fit parameters for two models using PET data from two different reference regions.

	T1 and T2						
**Reference region**	**Upregulation model**				**E_{max} model**		
	**EC_{50}**	**URF**	**AIC**	**RSE**	**EC_{50}**	**AIC**	**RSE**
Occipital Cortex (Defined *a priori*)	6.3	2.8	195	12.7	35	204	15.8
White Matter (Used *post hoc*)	11	1.9	178	9.0	31	180	9.2

Abbreviations: AIC, Akaike Information Criteria; RSE, residual standard error; URF, upregulation factor.

Supplementary Table 2. Test-Retest in 2 cynomolgus monkeys.

**Identification Numbers**	**A7701**			**A7702**		
**Region**	**Baseline BP_{ND} 1**	**Baseline BP_{ND} 2**	**%TRTV**	**Baseline BP_{ND} 1**	**Baseline BP_{ND} 2**	**%TRTV**
Caudate	4.16	3.90	6.6	4.83	4.93	-1.9
Putamen	6.37	5.85	8.4	6.33	6.44	-1.7

Abbreviations: %TRTV, percent test-retest variability; BP_{ND}, non-displaceable binding potential, measured in two independent baseline PET scans.

SUPPLEMENTARY REFERENCES

1 Tong J, Boileau I, Furukawa Y, Chang LJ, Wilson AA, Houle S, et al. Distribution of vesicular monoamine transporter 2 protein in human brain: implications for brain imaging studies. J Cereb Blood Flow Metab. 2011;31(10):2065-75.

2 Kilbourn MR, Cole EL, Scott PJH. In vitro binding affinity vs. in vivo site occupancy: A PET study of four diastereomers of dihydrotetrabenazine (DTBZ) in monkey brain. Nucl Med Biol. 2021;92:38-42.

3 Vander Borght T, Kilbourn M, Desmond T, Kuhl D, Frey K. The vesicular monoamine transporter is not regulated by dopaminergic drug treatments. Eur J Pharmacol. 1995;294(2-3):577-83.

4 Kilbourn MR, Frey KA, Vander Borght T, Sherman PS. Effects of dopaminergic drug treatments on in vivo radioligand binding to brain vesicular monoamine transporters. Nucl Med Biol. 1996;23(4):467-71.

5 Kilbourn MR, Butch ER, Desmond T, Sherman P, Harris PE, Frey KA. In vivo [11C]dihydrotetrabenazine binding in rat striatum: sensitivity to dopamine concentrations. Nucl Med Biol. 2010;37(1):3-8.

6 De La Fuente-Fernández R, Furtado S, Guttman M, Furukawa Y, Lee CS, Calne DB, et al. VMAT2 binding is elevated in dopa-responsive dystonia: visualizing empty vesicles by PET. Synapse. 2003;49(1):20-8.

7 Guo N, Guo W, Kralikova M, Jiang M, Schieren I, Narendran R, et al. Impact of D2 receptor internalization on binding affinity of neuroimaging radiotracers. Neuropsychopharmacology. 2010;35(3):806-17.

8 Liu H, Jin H, Luo Z, Yue X, Zhang X, Flores H, et al. In Vivo Characterization of Two (18)F-Labeled PDE10A PET Radioligands in Nonhuman Primate Brains. ACS Chem Neurosci. 2018;9(5):1066-73.

9 Ooms M, Attili B, Celen S, Koole M, Verbruggen A, Van Laere K, et al. [18F]JNJ42259152 binding to phosphodiesterase 10A, a key regulator of medium spiny neuron excitability, is altered in the presence of cyclic AMP. J Neurochem. 2016;139(5):897-906.
